# Effects of acute nutritional ketosis during exercise in adults with glycogen storage disease type IIIa are phenotype‐specific: An investigator‐initiated, randomized, crossover study

**DOI:** 10.1002/jimd.12302

**Published:** 2020-09-07

**Authors:** Irene J. Hoogeveen, Foekje de Boer, Willemijn F. Boonstra, Caroline J. van der Schaaf, Ulrike Steuerwald, Anita J. Sibeijn‐Kuiper, Riemer J. K. Vegter, Johannes H. van der Hoeven, M. Rebecca Heiner‐Fokkema, Kieran C. Clarke, Pete J. Cox, Terry G. J. Derks, Jeroen A. L. Jeneson

**Affiliations:** ^1^ Section of Metabolic Diseases, Beatrix Children's Hospital University of Groningen, University Medical Center of Groningen Groningen The Netherlands; ^2^ National Hospital of the Faroe Islands, Medical Center Tórshavn Faroe Islands; ^3^ Neuroimaging Center, Department of Neuroscience University Medical Center Groningen Groningen The Netherlands; ^4^ Center for Human Movement Sciences, University Medical Center Groningen University of Groningen Groningen The Netherlands; ^5^ Department of Neurology, University Medical Centre Groningen University of Groningen Groningen The Netherlands; ^6^ Department of Laboratory Medicine, Laboratory of Metabolic Diseases, University Medical Center Groningen University of Groningen Groningen The Netherlands; ^7^ Department of Physiology, Anatomy and Genetics University of Oxford Oxford UK; ^8^ Center for Child Development and Exercise, Wilhelmina Children's Hospital University Medical Center Utrecht Utrecht The Netherlands

**Keywords:** ^31^P‐MRS, acute nutritional ketosis, exercise, glycogen storage disease, ketone‐ester

## Abstract

Glycogen storage disease type IIIa (GSDIIIa) is an inborn error of carbohydrate metabolism caused by a debranching enzyme deficiency. A subgroup of GSDIIIa patients develops severe myopathy. The purpose of this study was to investigate whether acute nutritional ketosis (ANK) in response to ketone‐ester (KE) ingestion is effective to deliver oxidative substrate to exercising muscle in GSDIIIa patients. This was an investigator‐initiated, researcher‐blinded, randomized, crossover study in six adult GSDIIIa patients. Prior to exercise subjects ingested a carbohydrate drink (~66 g, CHO) or a ketone‐ester (395 mg/kg, KE) + carbohydrate drink (30 g, KE + CHO). Subjects performed 15‐minute cycling exercise on an upright ergometer followed by 10‐minute supine cycling in a magnetic resonance (MR) scanner at two submaximal workloads (30% and 60% of individual maximum, respectively). Blood metabolites, indirect calorimetry data, and in vivo ^31^P‐MR spectra from quadriceps muscle were collected during exercise. KE + CHO induced ANK in all six subjects with median peak βHB concentration of 2.6 mmol/L (range: 1.6‐3.1). Subjects remained normoglycemic in both study arms, but delta glucose concentration was 2‐fold lower in the KE + CHO arm. The respiratory exchange ratio did not increase in the KE + CHO arm when workload was doubled in subjects with overt myopathy. In vivo ^31^P MR spectra showed a favorable change in quadriceps energetic state during exercise in the KE + CHO arm compared to CHO in subjects with overt myopathy. Effects of ANK during exercise are phenotype‐specific in adult GSDIIIa patients. ANK presents a promising therapy in GSDIIIa patients with a severe myopathic phenotype.

**Trial registration number:**

ClinicalTrials.gov identifier: NCT03011203.

## Graphical abstract

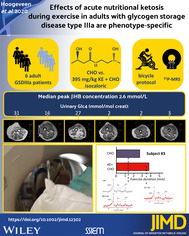

**Abbreviations**
31^P^‐MR: 31 phosphorus magnetic resonance
βHB: beta‐hydroxybutyrate
AcAc: acetoacetate
ANK: acute nutritional ketosis
COV: coefficient of variation
CPET: cardio‐pulmonary exercise test
FAO: fatty acid oxidation
FFAs: free fatty acids
GDE: glycogen debranching enzyme
GSD: glycogen storage disease
GSDIIIa: glycogen storage disease type IIIa
HMPs: hexose‐mono‐phosphates
KE: ketone‐ester
PCr: phosphocreatine
Pi: inorganic phosphate
RER: respiratory exchange ratio
RPE: rate of perceived exertion
RQ: respiratory quotient
VO_2_max: maximum oxygen uptake
Wmax: maximal workload

## 1 INTRODUCTION

Glycogen storage disease type IIIa (GSDIIIa; OMIM #232400) is an inborn error of carbohydrate metabolism caused by pathogenic variants in the *AGL* gene, resulting in impaired glycogen debranching enzyme (GDE) activity in liver, cardiac, nerve, and muscle tissue. According to the International Study on GSDIII (ISGSDIII), most patients present before the age of 1.5 years with various combinations of hepatomegaly, failure to thrive and fasting intolerance.^1^ Biochemically, the phenotype is characterized by fasting ketotic hypoglycemia, postprandial hyperlactatemia, increased transaminases, and hyperlipidemia.^2^

Dietary management to maintain normoglycemia and prevent hyperketonemia is the mainstay of treatment in GSDIIIa patients. Specifically, it involves designed dosing and frequency of a high‐protein diet with cornstarch supplementation.^3^, ^4^ However, despite such dietary management, 52% of patients report exercise intolerance and 31% suffer from proximal myopathy in an observational, international multicenter study of a relatively young patient cohort.^1^ Therefore, these percentages could even be an underestimation of the actual burden in adulthood. Moreover, progression of myopathy with age is observed by muscle ultrasound and dynamometry.^5^, ^6^ Although longitudinal studies are lacking, the available evidence suggests a shift from an acute, fasting‐intolerance‐associated hepatic phenotype in childhood toward a chronic, skeletal muscle, and hepatic phenotype in adult GSDIIIa patients.^7^, ^8^

The pathophysiology underlying muscle dysfunction in GSDIIIa patients is still incompletely understood. Various disease mechanisms have been proposed. First, the primary GDE deficiency together with high carbohydrate intake could cause excessive storage of an abnormal glycogen structure (ie, limit dextrin) in muscle interfering with contractile function.^9^, ^10^, ^11^ Second, increased endogenous proteolysis of skeletal muscle to provide adequate amino acids as gluconeogenic substrate to the liver could contribute to muscle wasting.^12^ Last, in vivo findings of delayed intramuscular metabolic recovery postexercise in a study in GSDIIIa patients suggest that myopathic symptoms may also result from cellular energy crisis during exercise as a result of reduced mitochondrial capacity for oxidative ATP synthesis.^13^

To date, physical training remains the most effective approach to prevent and reverse progressive loss of skeletal muscle mass and muscle quality.^14^ Any safe translation of this concept to the management of GSDIIIa patients is, however, severely complicated by the fact that GDE deficient muscles rely more on the metabolism of blood glucose than intramuscular glycogen for oxidative ATP synthesis.^15^ Moderate concentrations of ketone bodies beta‐hydroxybutyrate (βHB) and acetoacetate (AcAc) in the bloodstream may provide exercising muscles with an alternative external source of oxidative fuel than blood glucose.^16^ In 2012, an edible ketone‐ester ((R)‐3‐hydroxybutyl (R)‐3‐hydroxybutyrate; KE) for human application was described that can achieve acute nutritional ketosis (ANK) via oral ingestion without any sodium loading.^17^ In trained athletes, oral KE ingestion resulted in glycogen sparing during exercise, and a slight enhancement of endurance exercise performance and recovery.^18^, ^19^ Recently, oral KE ingestion prior to exercise was shown to be effective to deliver oxidative substrate to exercising leg muscle and improve intramuscular energy balance during submaximal cycling exercise in patients with a fatty acid oxidation (FAO) defect.^20^

Here, this matter was further investigated. Specifically, we investigated if ANK in response to KE ingestion is equally effective in adult patients with GSDIIIa to deliver oxidative substrate to exercising muscle with favorable effects on intramuscular energy balance state during submaximal exercise.

## 2 SUBJECTS AND METHODS

### 2.1 Study approval

The Medical Ethical Committee of the University Medical Center Groningen (UMCG), the Netherlands approved the study protocol (ref. no. METc2016.591). The study was conducted according to the principles of the Helsinki Declaration of 1975 as revised in 1983. All subjects provided written informed consent prior to inclusion in the study.

### 2.2 Subjects

Adults with GSDIIIa were recruited by the center of expertise for hepatic GSD at the UMCG, the Netherlands and the Faroes Hospital, Tórshavn, Faroe Islands. The trial was conducted at the UMCG between February 2017 and March 2018. Inclusion criteria were (a) confirmation of GSDIII with enzyme assay and/or *AGL* variation analysis, GSDIIIa further specified as GDE deficiency in muscle or clinical and/or biochemical signs of cardiac and/or skeletal muscle involvement, and (b) age from 18 to 65 years. Exclusion criteria included (a) pregnancy or breastfeeding, (b) insulin‐dependent diabetes mellitus, (c) recent cardiac disease (including cardiomyopathy, coronary artery disease, or a positive history for angina pectoris), (d) contraindications for magnetic resonance imaging studies, (e) unable to perform bicycle exercise, and (f) intercurrent illness which may influence exercise tolerance. Figure S2 presents the participant flow chart.

### 2.3 Study design

This was an investigator‐initiated, randomized, researcher‐blinded, comparator‐controlled, two‐way crossover study (NCT03011203). Three consecutive study visits were scheduled at the UMCG, after written informed consent. Foreign subjects stayed in a hotel close to the study site during the whole study period. Other subjects stayed in the hotel the night before study visit 2 and 3.

### 2.4 Procedures

Figure S1 presents the study protocol.

#### 2.4.1 Study visit 1: Screening visit

General history, physical examination, muscle ultrasound, dynamometry and plasma analysis of liver transaminases, total creatine kinase, and NT‐proBNP were performed. The activity level was assessed by the International Physical Activity Questionnaire.^21^ Muscle ultrasound and dynamometry were performed and analyzed as described previously.^6^ *Z*‐scores for muscle ultrasound density of the biceps, quadriceps, calf (gastrocnemius and/or soleus), and tibialis anterior muscles were calculated based on age‐related references values.^22^ After at least 2 hours of rest subjects performed a cardio‐pulmonary exercise test (CPET) to determine subjects' individual maximal workload (Wmax) and maximum oxygen uptake (VO_2_max).

#### 2.4.2 Study visit 2/3: Exercise protocol with prior ingestion of study drink

Subjects fulfilled an identical exercise protocol during visits 2 and 3, which were separated by 7 days in all subjects. Subjects were asked to refrain from alcohol and caffeine for 24 hours prior to each study visit and to consume a similar breakfast in the morning of both study visits. A 3‐day food diary was collected prior to visit 2 and 3. At 8:00 am, a taxi brought the subject to the study site. Here the subject was transported in a wheelchair to minimize exercise before study procedures. After general instructions and positioning for exercise, the subject was given the study drink at approximately 9:00 am (*t* = 0). Forty‐five minutes after study drink ingestion, the subject started with a 15‐minute upright bicycle protocol. The target pedaling frequency was 70 rounds per minute (rpm). During the upright bicycle protocol, indirect caloric and heart rate measurements were collected (Cosmed K4, Lode Excalibur). Ratings of perceived exertion (RPE) were assessed with the Borg scale.^23^ After the 15‐minute upright bicycle protocol, subjects started 10 minutes of cycling inside the MR scanner. In each exercise bout, workload was increased from 30% to 60% of the subject's individual Wmax for the last 5 minutes. Blood was sampled via an intravenous catheter at baseline, during upright bicycle exercise, during supine exercise inside the MR scanner, and 3 hours after exercise. Samples were directly analyzed for βHB, AcAc, glucose, insulin, lactate, and free fatty acids (FFAs) by standard laboratory procedures. Urine was collected in the time between study drink ingestion and until 3 hours after exercise.

### 2.5 Outcome measures

The primary outcome measures were blood βHB and glucose concentrations, exercise performance, as assessed with indirect caloric and heart rate measurements, and 31 phosphorus magnetic resonance (^31^P‐MR) spectra during exercise and recovery. The ^31^P‐MR spectroscopy permits continuous and noninvasive monitoring of inorganic phosphate (Pi), phosphocreatine (PCr), and pH, allowing assessment of muscle energy metabolism during exercise.^24^ Secondary outcomes were blood concentrations of AcAc, insulin, lactate, and FFAs, RPE scores, and urinary excretion of βHB and glucose tetrasaccharide (Glc4). Glc4 was analyzed by LC‐MS/MS according to,^25^ with minor adjustments.^25^

### 2.6 Investigational product

Study drinks were prepared at the study site 1 hour before ingestion. Subjects received 395 mg/kg of KE + 30 g maltodextrin (KE + CHO) or an isocaloric carbohydrate drink containing only maltodextrin ~66 g (CHO). In both study arms, a minimum of 1.2 g of carbohydrate per minute exercise supply was ensured.^26^, ^27^

### 2.7 Randomization and blinding

Subjects were randomly assigned to a study drink order based on enrollment. The researcher (J. A. L.J.) who analyzed the ^31^P‐MRS data was blinded for study drink randomization. Ingestion and preparation of study drink took place in another study room to guarantee blinding for this researcher. All data sets from ^31^P‐MR spectra were coded for blinded analysis by one researcher (I. J. H.).

### 2.8 ^31^P‐MRS analysis

#### 2.8.1 ^31^P‐MRS data acquisition

In vivo ^31^P‐MR spectroscopic data on quadriceps energy and pH balance at rest, during exercise and postexercise were collected using a 3.0 Tesla whole‐body MR‐scanner fitted with a supine cycle ergometer (*Achiva*; *Philips Healthcare, Best, The Netherlands*) and analyzed according to methods described elsewhere.^28^ Dynamic acquisition of ^31^P‐MR spectra during 10‐minute cycling exercise at 70 to 80 rpm was synchronized with motion using custom‐built ergometer‐spectrometer interfacing hardware and software as described elsewhere.^29^ The brake‐weight required for workload equivalents of 30% and 60% of Wmax, respectively, was calculated for each subject as described elsewhere.^30^

#### 2.8.2 ^31^P‐MRS data processing

Data were processed and analyzed in the time domain using the AMARES algorithm in the public jMRUI software environment (version 3.0) in combination with prior knowledge information on ATP metabolite content and ^31^P‐MR spectral properties as described elsewhere (see also Figures S3‐S5).^29^ Intramuscular pH was determined from the resonance frequency of Pi using standard methods.^29^ Postexercise kinetics of Pi recovery to resting levels were analyzed by nonlinear curve‐fitting of a monoexponentially function yielding a fitted estimate of recovery time constant (in seconds) as described elsewhere.^29^

### 2.9 Statistical analysis

Data were analyzed using SPSS Statistics version 23.0 (IBM Corp., Armonk, New York) and visualized using Prism 5 software (GraphPad Software, Inc., La Jolla, California). Data from indirect calorimetry were processed using Matlab version 2019a (MathWorks, Inc., Natick, Massachusetts). A linear mixed model was used to analyze the effect of study drink on blood metabolites. Fixed effects in this model were the main effects of study arm, time, workload, and order of the study drinks in the cross‐over design as well as the two‐way interactions between study arm and workload and study arm and time and the three‐way interaction between study arm, time, and workload. Subject ID was included in the model as a random effect. Post hoc contrast analyses were performed to determine the effect of study drink per time point. Descriptive statistics were used for remaining outcome parameters and a two‐tailed paired Student's test was used for statistical differences in ^31^P‐MRS data. Data were considered statistically significant at *P* < .05.

## 3 RESULTS

### 3.1 Subjects

Six GSDIIIa (4F, 2 M) patients from four different countries were enrolled with a median age of 46 years (range: 36‐63). Table 1 presents the characteristics of study subjects. The outcomes of muscle ultrasound, dynamometry, and CPET showed a severe myopathic phenotype in subject #1, 2, and 3. Subject #2 presented with a lower leg support device to stabilize his right foot, while subject #3 needed a companion for walking support. MR images of the upper legs showed severe muscle atrophy and fat replacement in subject #1 and #3. In contrast, subjects #4, 5, and 6 had normal muscle tests and CPET outcomes. Also, urinary Glc4 concentrations were markedly lower in these subjects. Due to this large heterogeneity between subjects, results will be presented in two groups or individually. Group 1 includes subjects #1, 2, and 3 with overt myopathy, and group 2 includes subjects #4, 5, and 6 without overt myopathy.

**TABLE 1:** Clinical and biochemical characteristics of subjects

	1	2	3	4	5	6
*General*						
Age range (y)	36	36‐40	46‐50	61‐65	46‐50	56‐60
BMI (kg/m^2^)	24.2	30.7	30.4	28.8	30.8	29.2
Molecular defect *AGL* gene						
Nucleotide change	c.4529dupA c.4529dupA	c.765G>A c.4529dupA	c.2590C>T c.3247delT	c.1222C>T c.1222C>T		
*Dietary management*						
E%, carbohydrates	21%	42%	22%	28%	22%	39%
E%, protein	13%	17%	27%	47%	34%	27%
*Muscle status*						
History of muscle weakness	Proximal lower extremities	Distal upper and lower extremities	Proximal lower extremities	Distal upper extremities	Proximal, distal, lower and upper extremities	Distal lower extremities
Blood markers						
ASAT (U/L)	**100**	**158**	**128**	**35**	29	**48**
ALAT (U/L)	**86**	**108**	**138**	32	34	**68**
NT‐proBNP (ng/L)	125	<5	89	133	92	104
Total CK (U/L)	**904**	**3442**	**957**	**174**	102	107
*Urinary Glc4 (mmol/mol creat)*	31	16	27	2	2	3
MUD *Z*‐scores quadriceps	+3.88	+1.94	+3.76	−0.25	+0.35	+0.05
MR imaging of quadriceps muscle	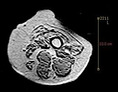	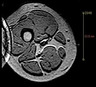	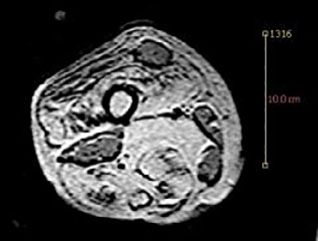	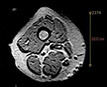	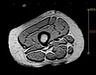	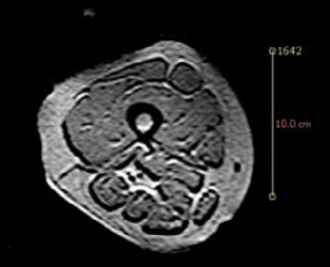
*Muscle strength and exercise*						
Activity level^a^	Moderate	Moderate	Moderate	Moderate	High	High
Dynamometry	Tetra paresis	Distal paresis	Proximal paresis	Normal	Normal	Normal
VO_2_ max (% of predicted)	52	58	46	95	105	96
W_max_ (% of predicted)	34	36	24	138	148	130

*Note:* The values in bold indicate above local laboratory reference values.

Abbreviations: AGL, amylo‐α‐1,6‐glucosidase 4‐α‐glucanotransferase; ALAT, alanine aminotransferase; ASAT, aspartate aminotransferase; CK, creatine kinase; E%, energy percentage of total caloric intake; Glc4, glucose tetrasaccharide; MUD, muscle ultrasound density; NT‐proBNP, N‐terminal prohormone of brain natriuretic peptide; VO_2_ max, maximal oxygen uptake; Wmax, maximal workload.

^a^ Based on international physical activity questionnaire.^21^

### 3.2 Tolerance of KE and ANK

KE was well tolerated by all subjects. One subject (#2) reported mild headache after ingestion of KE + CHO (maximum βHB concentration in this patient reached 2.8 mmol/L). The other subjects did not report any symptoms of nausea, headache, or stomach pain after ingestion of the KE + CHO drink. No adverse events were reported.

### 3.3 Effect of ANK on blood and urine metabolites

**FIGURE 1:**
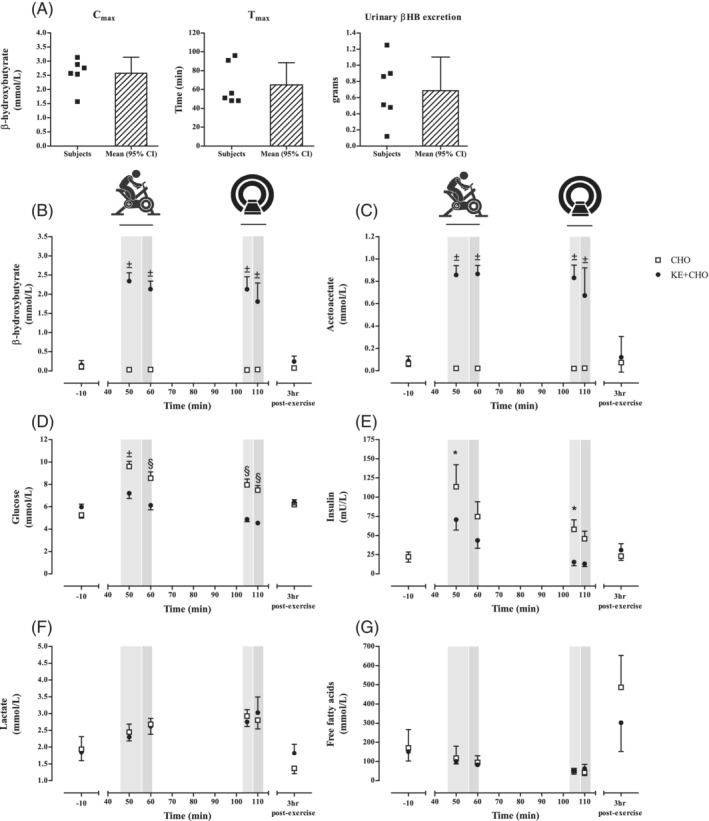
Changes of blood and urine metabolites after ingestion of either carbohydrates (CHO) or carbohydrates and ketone‐ester (KE + CHO) drink before, during, and after exercise. A, βHB kinetics after ingestion of KE + CHO; B, βHB concentrations; C, AcAc concentrations; D, glucose concentrations; E, insulin concentrations; F, lactate concentrations; and G, FFA concentrations. In panel (B‐G), n = 4 for time points *t* = 105 and *t* = 110 (during in‐magnet exercise), n = 6 for all other time points in both study arms. Light gray columns represent the time frame of exercise at 30% Wmax, dark gray columns represent the time frame of exercise at 60% Wmax. Values expressed as mean ± SEM. *P < .05, ^§^ *P* < .01, ^±^ *P* < .0001; linear mixed model analysis with post hoc contrast analysis. βHB, beta‐hydroxybutyrate; FFA, free fatty acid

Figure 1 presents the concentration kinetics of selected blood metabolites throughout the study protocol in both study arms. Ingestion of KE + CHO induced significant ANK within 1 hour (Figure 1A,B). Peak βHB and AcAc concentrations were on average 2.6 mmol/L (range: 1.6‐3.1) and 1.0 mmol/L (range: 0.7‐1.2), respectively (Figure 1A,C). Median βHB concentrations at *t* = 0 ranged from 0.0 to 0.4 mmol/L in the CHO arm and from 0.0 to 0.7 mmol/L in the KE + CHO arm. Four hours after ingestion of KE + CHO median βHB concentration was 0.5 mmol/L (n = 5, range: 0.1‐0.8). All subjects remained normoglycemic in both study arms (glucose concentrations >3.6 mmol/L, Figure 1D), but glucose concentrations were higher throughout the exercise protocol after ingestion of CHO vs KE + CHO (*t* = 50; *P* < .0001, *t* = 60, *t* = 105, *t* = 110; *P* < .01; linear mixed model, Figure 1D). The average delta of glucose concentrations was almost 2‐fold higher in the CHO arm vs the KE + CHO arm, specifically 4.7 mmol/L vs 2.6 mmol/L (Table S1). Workload did not affect glucose concentrations differently between study arms. Insulin concentrations were lower at *t* = 50 and *t* = 105 in the KE + CHO arm (*P* < .05; linear mixed model, Figure 1E). Lactate concentrations increased from baseline into exercise, but there were no differences between study arms at different timepoints (*P* > .05; linear mixed model, Figure 1F). FFAs in blood remained low throughout the study protocol in both arms (Figure 1G) and were influenced by lunch 3 hours postexercise. Urinary myoglobin concentrations were within the local reference range (<21 μg/L) in both study arms in five out of six subjects. In subject #1, urinary myoglobin concentration was slightly increased after KE + CHO ingestion, namely 34 μg/L, but not after CHO. No symptoms or signs of acute rhabdomyolysis were reported by the study subjects during the phone calls the day after study visit 2 and 3.

### 3.4 Effect of ANK on cardiorespiratory parameters during exercise

**FIGURE 2:**
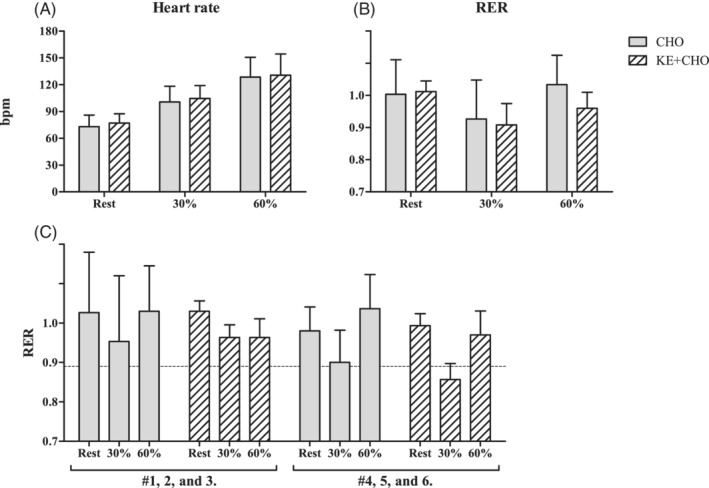
Heart rate and indirect calorimetry measurements at rest, 30% Wmax, and 60% Wmax during the upright bicycle protocol in both study arms. A,B, Pooled data (n = 6); C, Data presented as subgroups based on muscle phenotype, n = 3 in both groups. Dashed line represents the RQ of βHB (0.89). Data presented as mean ± SD. βHB, beta‐hydroxybutyrate; RQ, respiratory quotient

Figure 2 shows the results of heart rate and indirect caloric measurements in both study arms of all subjects (n = 6). Median (range) RPE scores were 7 (6‐9) and 7 (6‐8) at 30%Wmax, and 9 (7‐13) and 10 (7‐14) at 60%Wmax with and without ANK, respectively. Heart rate increased on average from 70 at rest (upright position on ergometer) to 100 bpm at 30% Wmax to 130 bpm at 60% Wmax in both study arms (Figure 2A). The respiratory exchange ratio (RER) was 1.0 at rest and decreased to 0.8 to 0.9 during exercise at 30% Wmax in both arms (Figure 2B, Table S2). During exercise at 60% Wmax, RER went back up to 1.0 only in the CHO arm (Figure 2B). Comparing measured RER values during exercise between overt (#1, 2, and 3) and nonovert myopathic subjects (#4, 5, and 6), no difference was found in the CHO arm (Figure 2C). However, in the KE + CHO arm, RER seems to decrease more from rest to 30% Wmax in nonovert myopathic subjects than in overt myopathic subjects (Figure 2C). Specifically, RER during exercise at 30%Wmax in nonovert myopathic patients was 0.86 compared to 0.96 in overt myopathic patients (Figure 2C). In the KE + CHO arm, RER did not change when workload was increased from 30% to 60% Wmax in subjects with overt myopathy. The coefficient of variation (COV; SE/mean) of RER was 2‐ to 3‐fold lower in the KE + CHO arm than in the CHO arm in both groups (Figure 2C).

### 3.5 Effect of ANK on in vivo quadriceps energy balance during cycling exercise

All subjects without overt myopathy (subjects #4, 5, and 6) completed the supine cycling exercise task inside the MR scanner. Due to technical difficulties, the data of the CHO arm collected during exercise could not be analyzed for subject #5. Of the three subjects with overt myopathy (subjects #1, 2, and 3), only subject #1 was able to complete the regular in‐magnet exercise task. Subject #3 performed an adapted exercise task consisting of propelling the ergometer flywheel without any mechanical braking (“idle” resistance of the ergometer) due to insufficient leg muscle power. Subject #2 was unable to perform any form of supine cycling exercise in the MR scanner due to foot flexor paralysis.

**FIGURE 3:**
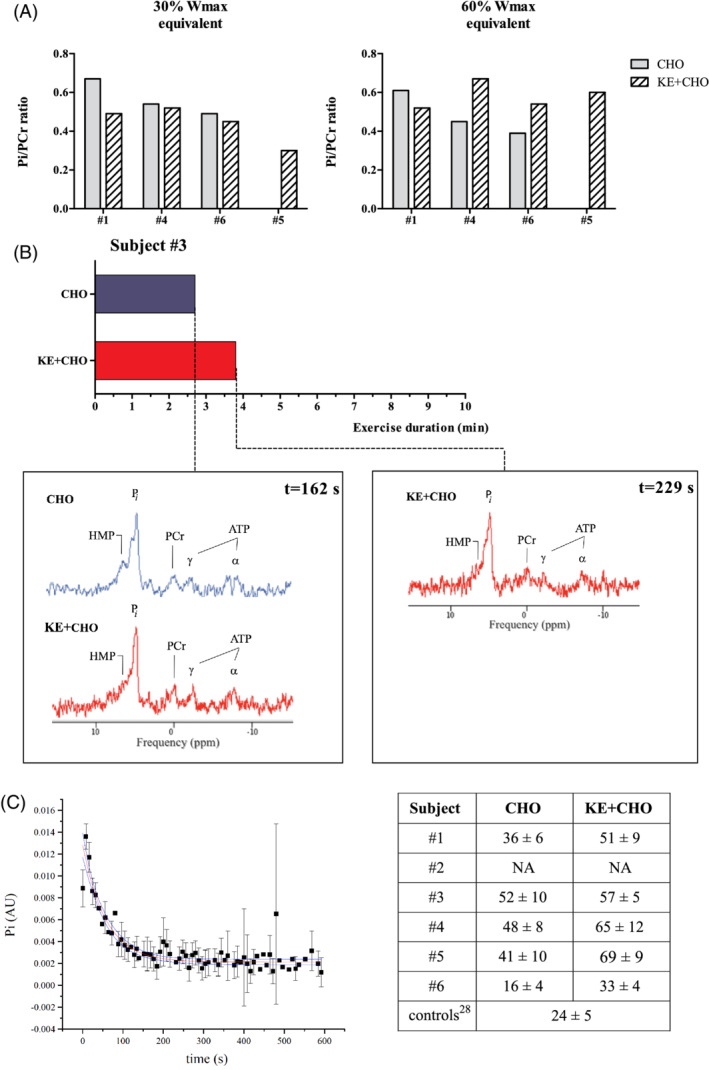
Outcomes of in vivo 31^P^‐MR spectra of quadriceps muscle during 10‐minute supine in‐magnet exercise and recovery in both study arms. A, Intramuscular Pi/PCr ratios at equivalents of 30% and 60% Wmax in four subjects; B, Exercise duration and related spectra, in both study arms for subject #3; C, Example of intramuscular Pi recovery time course from subject #3 in the KE + CHO arm (left panel), table represents individual rates of metabolic recovery vs healthy controls^30^ (right panel). 31^P^‐MR, 31 phosphorus magnetic resonance; CHO, carbohydrates; KE, ketone‐ester; PCr, phosphocreatine; Pi, inorganic phosphate

Pi/PCr ratios are useful measures of muscle mitochondrial function, where a decrease in Pi/PCr ratio reflects improved mitochondrial efficacy. Figure 3A shows the measured Pi/PCr ratio in the quadriceps muscle of subject #1, #4, #6, and #5 during exercise at two submaximal workloads in both study arms. At 30% Wmax, the Pi/PCr ratio measured in the presence of ANK was lower than in the CHO arm in three subjects (subject #1, 4, and 6; Figure 3A, left panel). At 60% Wmax, quadriceps Pi/PCr ratio measured in the presence of ANK in subject #1 was likewise lower than in the CHO arm, but not in subjects without overt myopathy (subjects #4 and 6; Figure 3A, right panel). Mild muscle alkalosis was observed during exercise at both workloads in subject #1 in both study arms. In subject #4, this was found only in the CHO arm (Table S3).

Figure 3B shows the results of the in vivo ^31^P‐MR measurements in quadriceps muscle during in‐magnet cycling exercise for subject #3. In the CHO arm, this subject was able to maintain cycling exercise for 162 seconds (Figure 3B). The ^31^P‐MR spectrum of this patient at exhaustion showed that the intramuscular PCr store was almost completely depleted concomitant with millimolar accumulation of hexose‐monophosphates (HMPs) in contracting fibers (Figure 3B). In the KE + CHO arm, the patient was able to maintain cycling exercise for 229 seconds—that is, 67 seconds longer than in the CHO arm (Figure 3B). The ^31^P‐MR spectrum of the quadriceps muscle at exhaustion in the KE + CHO arm was almost identical to the ^31^P‐MR spectrum obtained at 162 seconds of cycling in the CHO arm (Figure 3B) except for two particulars: (a) the amplitude of the HMP signals at exhaustion in the presence of ANK was lower than in the CHO arm (Figure 3B); (b) muscle pH at exhaustion was mildly alkalotic in the presence of ANK compared to mildly acidic in the CHO arm (Table S4, Figure S3‐S5).

### 3.6 Effect of ANK on postexercise metabolic recovery kinetics

Figure 3C shows a typical example of time course of intramuscular Pi immediately following exercise. In four out of five subjects, the rate of metabolic recovery, indexed by the time constant *tau* of Pi recovery toward resting level (τPi; in seconds), was almost 2‐fold slower than previously reported for healthy human quadriceps muscle (Figure 3C, table).^30^ Within the accuracy of τPi estimation, there were no individual differences in rate of metabolic recovery between study arms.

## 4 DISCUSSION

This study in six adults with GSDIIIa investigated whether ANK in response to oral ingestion of a KE can supply oxidative substrate to exercising muscle. ANK was efficiently induced within 1 hour after ingestion of KE + CHO, KE was well tolerated, and improved glucose homeostasis. We obtained in vivo evidence that ANK has a beneficial effect on muscle energy balance during exercise in GSDIIIa patients with a severe muscle phenotype. In patients without any overt muscle phenotype, we found no beneficial effect on muscle energy balance.

In the present study, the ingestion of 395 mg/kg KE in subjects with GSDIIIa resulted in ANK with maximum βHB concentrations (1.6‐3.1 mmol/L) comparable to those previously found in healthy adults^18^, ^31^ and patients with Very Long‐Chain acyl‐CoA Dehydrogenase deficiency (VLCADD).^20^ Subjects remained normoglycemic in the KE + CHO arm during the entire protocol. Furthermore, the delta in glucose concentration was almost 2‐fold lower than in the eucaloric CHO arm with related lower insulin concentrations. The latter may well have been the direct result of the 2‐fold higher maltodextrin intake in the CHO study arm. This amount of CHO supplementation (~66 g) was comparable to a previous fructose supplementation study in GSDIIIa patients.^32^

The whole‐body indirect calorimetry results confirmed that subjects performed exercise at submaximal workloads, with peak heart rates around 130 bpm at the highest imposed workload. When stratifying for muscle phenotype, a striking finding was that the COV was 2‐ to 3‐fold lower in the KE + CHO arm compared to the CHO arm in both groups—that is, subjects with overt myopathy (#1‐3) and subjects without overt myopathy (#4‐6) (Figure 2C). On a whole body level, ANK was associated with a more consistent metabolic state than CHO alone. The particular trend observed in the CHO arm in both groups, fitted well with the “cross over” concept of whole‐body oxidative substrate utilization during incremental exercise—that is, predominantly fatty‐acid oxidation at workloads below 40%Wmax progressively shifting toward CHO oxidation at higher workloads.^33^ In subjects without overt myopathy, this trend in RER was also observed in the KE + CHO arm. In subjects with overt myopathy, however, RER did not increase with workload change from 30% to 60% suggesting incomplete non‐CHO substrate utilization. This could be either βHB (RQ 0.9) or a mix of fat (RQ 0.7) and βHB.^18^

Complete data sets on in vivo energy and pH balance in exercising quadriceps muscle in both study arms were obtained in three subjects. In vivo intramuscular Pi/PCr ratios during exercise at the lowest workload in each arm suggested that leg muscle of these subjects used ketones as oxidative substrate in the KE + CHO arm. Previously, Kim et al found a small reduction of in vivo Pi/PCr ratio of the myocardium in dogs infused with βHB compared to control.^34^ In subject #1, a relatively large reduction in Pi/PCr ratio in the KE + CHO compared to CHO arm was observed at both 30% and 60%Wmax equivalents (Figure 3A). It is unlikely that this was solely the result of improved thermodynamic efficiency of oxidative ATP synthesis by ketone oxidation. Rather it may well reflect that recruitment of fewer motor units was needed to perform the voluntary exercise task during ANK due to improved work efficacy.^35^ Indeed, subject #3 was able to perform the same voluntary exercise task almost 1 minute longer in the KE + CHO arm than in the CHO arm. The in vivo ^31^P spectrum at exhaustion recorded in the CHO arm showed large accumulation of phosphorylated glycolytic intermediates as well as mild muscle acidification, both of which were absent in the KE + CHO arm (Figure 3B). In subjects #4 and #6, we did not find any favorable effect of ANK on muscle energy balance during exercise at the highest submaximal workload. Last, ANK did not have any effect on postexercise metabolic recovery kinetics (Figure 3C) similar to previous findings in VLCADD patients.^20^ This was an expected outcome as it has previously been shown that these kinetics are independent of end‐exercise state of muscle energy balance for low‐to‐moderate exercise workloads.^36^ However, the postexercise recovery time of Pi in quadriceps muscle of the subjects was on average 2‐fold slower than previously reported in healthy controls (Figure 3C, table). This result was in close agreement with previous ^31^P MRS findings in calf muscle of GSDIII patients^13^

On basis of these results, we conclude that ANK during exercise induced by prior KE ingestion may be beneficial to GSDIIIa patients when engaging in physical activity. Specifically, the results of this study suggest that such therapeutic approach should principally be focused on patients with a severe muscle phenotype, exemplified by subjects #1 to 3 in this study. Nevertheless, long‐term follow‐up studies are needed in more patients to assess efficacy and safety. Here it may be important to note that subjects #4 to 6 all originated from and resided in the same North‐Atlantic archipelago with a known founder pathogenic variation,^37^ whereas subjects #1 to 3 all originated from different countries in Europe. This prompts consideration of genetic and environmental modifying factors contributing to the observed differences in muscle phenotype in GSDIIIa. Subjects #1 to 3 carry unique nonsense *AGL* genotypes which involves at least one duplication or deletion, whereas the homozygous nonsense single‐base substitution c.1222C>T (R408X) *AGL* genotype in subjects #4 to 6 causes truncation of enzyme, which affects both enzymatic functions, namely oligo‐1,4‐1,4‐glucanotransferase and amylo‐1,6‐glucosidase.^37^ It is therefore likely that additional genetic or dietary factors may explain the phenotypes. Interestingly, average daily protein intake of subjects #4 to 6 was up to 2‐fold higher than reported by subjects #1 to 3 (Table 1). A recent study in *AGL* knock‐out mice demonstrated a reduction in muscle wasting in mice fed a high protein and glucose restricted diet.^11^ Various case studies have also demonstrated a reversal of myopathy defined by increased physical strength and reduced CK concentrations after dietary interventions with high protein^10^, ^38^ and/or ketogenic diets.^39^, ^40^ These studies report different outcome measures and macronutrient distributions; hence, it remains an enigma whether muscle atrophy in adult GSD IIIa patients can be prevented by dietary interventions.

The generalizability of our findings is subject to several limitations. Like other clinical studies in patients with ultra‐rare disease, this study was complicated by difficulties of including sufficient subjects. Despite the demanding study protocol, we were able to recruit six patients from four different countries, reflecting the wide spectrum of clinical heterogeneity between adult GSDIIIa patients. The latter prompted the analysis of two n = 3 subgroups rather than one n = 6 population. Due to this small number of subjects and the great heterogeneity between individual GSDIIIa patients, definitive conclusions on the efficacy of ANK cannot be drawn for the whole cohort. The intervention was constrained by the absence of a negative control group because of the requirement of a “sufficient” amount of CHO in both study arms to ensure patient safety. This issue was discussed during a focus group meeting with patients, resulting in a decision to have safety arguments outweigh methodological arguments. Similarly, muscle biopsy was offered as an optional procedure in our protocol, similar to Cox^18^ and Bleeker.^20^ However, cross‐sectional MR images of the upper leg showed that any chance of successful sampling of muscle tissue from the leg of subjects #1 and #3 by non‐guided transcutaneous needle biopsy would be slim (Table 1). Of the four remaining subjects, only one subject (#2) gave informed consent. Last, although the subjects exercised with increased plasma concentrations of glucose (CHO) and ketones (KE + CHO), we cannot exclude that differences in absorption and requirement of maltodextrin vs KE in GSDIIIa patients may have caused different maximum plasma concentrations.

For decades, several descriptive studies have underlined the importance of investigation of muscle involvement in GSDIIIa patients,^1^, ^9^, ^41^, ^42^ besides progressive liver disease.^7^ Prevention and, if possible, reversal of progressive loss of skeletal muscle mass and quality in GSDIIIa patients is therefore a key objective in clinical management. Current guidelines on GSDIII management do not provide recommendations regarding exercise or pre‐exercise therapy^4^ but do mention the potential beneficial effect of aerobic conditioning as seen in McArdle's disease (GSDV; OMIM #232600).^43^ The recent international GSD priority setting partnership has added muscle problems to the list of research priorities for GSD patients.^44^ Valayannopoulos et al reported successful treatment of sodium‐D,L‐3‐hydroxybutyrate up to 800 mg/(kg d), in conjunction with a ketogenic and high‐protein diet, in a 2‐month‐old infant with GSD IIIa, complicated by severe cardiomyopathy.^45^ We recently reported decreased creatine kinase concentrations and a decrease in cardiac hypertrophy in pediatric GSDIIIa patients after the introduction of high fat diets.^46^ The current study of oral KE supplementation on in vivo muscle biochemistry and function in GSDIIIa patients provides a subsequent steppingstone toward translation of the theoretical beneficial effect of ANK to a pre‐exercise skeletal muscle therapy in selected, myopathic GSDIIIa patients. Furthermore, ANK with oral supplementation is less demanding than a restrictive, ketogenic diet. As such, we propose to study acute delivery of ketones as alternative to acute glucose or fructose supplementation^15^, ^32^ to support physical activity in this subgroup of GSDIIIa patients. Strict patient‐to‐patient interventions and long‐term monitoring of muscle status together with liver function and morphology are recommended in case of frequent use of KE to induce ANK.^47^

## CONFLICT OF INTEREST

The intellectual property and patents covering the uses of ketone bodies and esters are owned by BTG Ltd., The University of Oxford, the NIH and TdeltaS Ltd. Should royalties ever accrue from these patents, Kieran C. Clarke and Pete J. Cox as named inventors may receive a share of royalties as determined by the terms of the respective institutions. Kieran C. Clarke is director of TdeltaS Ltd., a spin out company of the University of Oxford, to develop and commercialize products based on the ketone‐ester. Irene J. Hoogeveen, Foekje de Boer, Willemijn F. Boonstra, Caroline J. van der Schaaf, Riemer J. K. Vegter, Johannes H. van der Hoeven, M. Rebecca Heiner‐Fokkema, Terry G. J. Derks, and Jeroen A. L. Jeneson declare that the research was conducted in the absence of any commercial or financial relationships that could be construed as a potential conflict of interest.

## ETHICS STATEMENT

The Medical Ethical Committee of the University Medical Center Groningen (UMCG), the Netherlands approved the study protocol (ref. no. METc2016.591). The study was conducted according to the principles of the Helsinki Declaration of 1975 as revised in 2000. All subjects provided written informed consent prior to inclusion in the study.

## DOCUMENTATION CARE AND USE OF LABORATORY ANIMALS

Not applicable.

## Supporting information


**Figure S1** Study protocol
**Figure S2.** Participant flow chart
**Figure S3‐5.** AMARES ^31^P‐MRS fitting details of subject #3.
**Table S1.** Changes in blood metabolites after ingestion of CHO and KE + CHO study drink from all subjects (n = 6).
**Table S2.** Individual data on RER during the upright bicycle protocol in both study arms.
**Table S3**. Individual data on muscle pH at 30% and 60% Wmax equivalents in both study arms.
