# Supplementary material for: Effects of acute nutritional ketosis during exercise in adults with glycogen storage disease type IIIa are phenotype‐specific: An investigator‐initiated, randomized, crossover study
Source: J Inherit Metab Dis. 2020 Sep 7;44(1):226–39. doi: 10.1002/jimd.12302 (PMC7891643; doi:10.1002/jimd.12302)
Supplement: Supplementary file 1 — Figure S1 Study protocol Figure S2. Participant flow chart Figure S3‐5. AMARES 31P‐MRS fitting details of subject #3. Table S1. Changes in blood metabolites after ingestion of CHO and KE + CHO study drink from all subjects (n = 6). Table S2. Individual data on RER during the upright bicycle protocol in both study arms. Table S3. Individual data on muscle pH at 30% and 60% Wmax equivalents in both study arms. [file JIMD-44-226-s001.docx]

*Supplementary Material*

Effects of acute nutritional ketosis during exercise in adults with glycogen storage disease type IIIa are phenotype-specific:

an investigator-initiated, randomized, crossover study

Irene J. Hoogeveen, MD^1^, Foekje de Boer, RD^1^, Willemijn F. Boonstra, BSc^1^,

Caroline J. van der Schaaf, RN^1^, Ulrike Steuerwald, MD^2^, Anita J. Sibeijn-Kuiper, MSc^3^, Riemer J. K. Vegter, PhD^4^, Johannes H. van der Hoeven, MD, PhD^5^,

M. Rebecca Heiner-Fokkema, PhD^6^, Kieran C. Clarke, MD, PhD^7^, Pete J. Cox, MD, PhD^7^, Terry G.J. Derks, MD, PhD^1*#^, Jeroen A.L. Jeneson, PhD^3#^.

^1^Section of Metabolic Diseases, Beatrix Children’s Hospital, University of Groningen, University Medical Center of Groningen, Groningen, The Netherlands

^2^National Hospital of the Faroe Islands, Medical Center, Tórshavn, Faroe Islands

^3^Neuroimaging Center, Department of Neuroscience, University Medical Center Groningen, Groningen, The Netherlands;

^4^Center for Human Movement Sciences, University Medical Center Groningen, University of Groningen, Groningen, The Netherlands;

^5^Department of Neurology, University Medical Centre Groningen, University of Groningen, The Netherlands;

^6^Department of Laboratory Medicine, Laboratory of Metabolic Diseases, University Medical Center Groningen, University of Groningen, Groningen, The Netherlands;

^7^Department of Physiology, Anatomy and Genetics, University of Oxford, Oxford, United Kingdom.

# Equal senior author

***Corresponding author**

Terry G.J. Derks, E-Mail: [t.g.j.derks@umcg.nl](mailto:t.g.j.derks@umcg.nl)

**Figure S1.** Study protocol

**
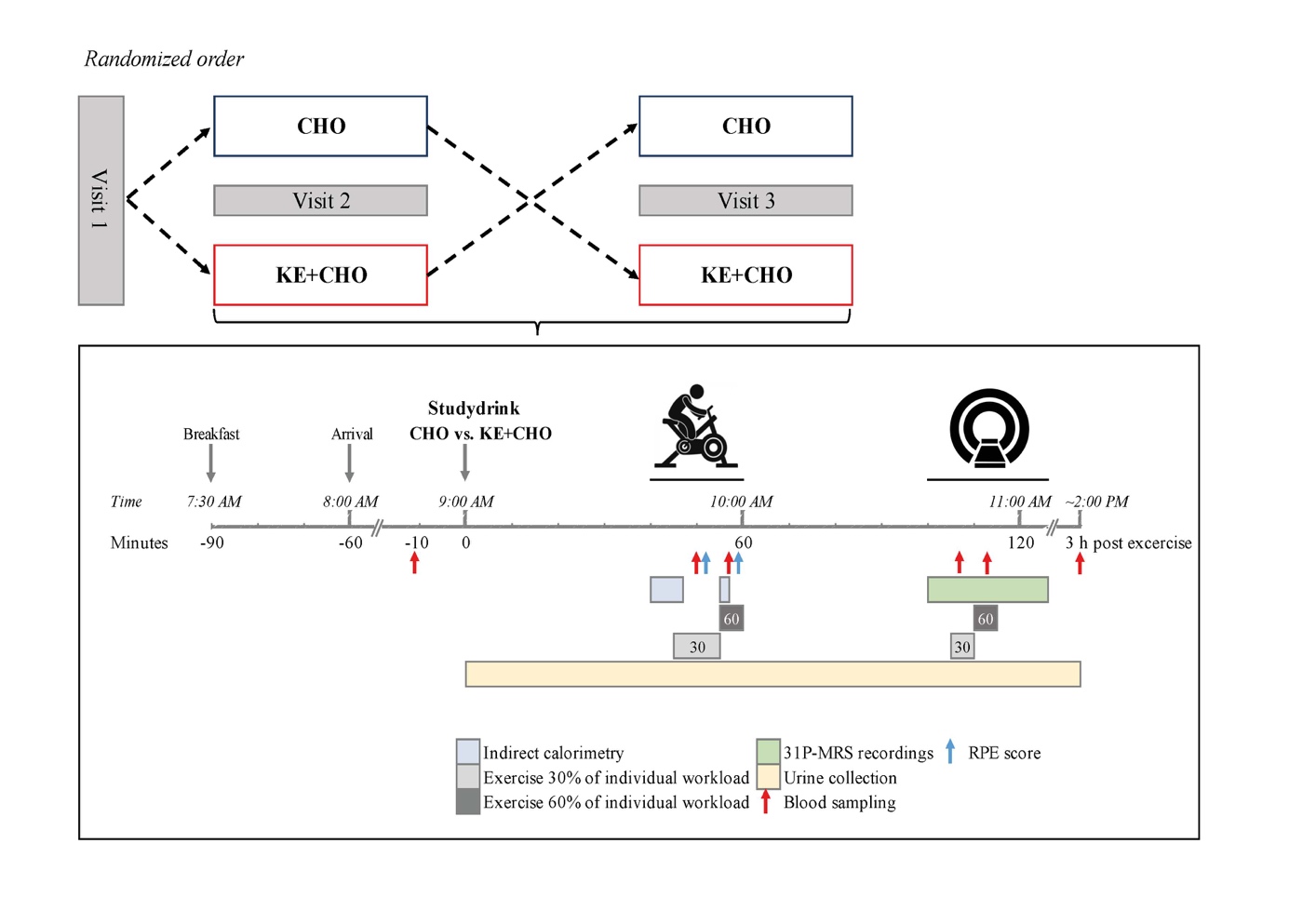
**

Legend: CHO, carbohydrate; KE, ketone-ester; 31P-MRS, ^31^Phosphorus magnetic resonance spectroscopy; RPE, rate of perceived exertion.

**Figure S2.** Participant flow chart


**Figure S3-5.** AMARES ^31^P-MRS fitting details of subject #3.

|  |  | | *Upright bicycle protocol* | | | | *Supine exercise inside MR scanner* | | | | *3 h post-exercise* | |
| --- | --- | --- | --- | --- | --- | --- | --- | --- | --- | --- | --- | --- |
|  | **T=0** | | **T=50** | | **T=60** | | **T=105** | | **T=110** | | **T= 295** | |
| **Study arm** | **CHO** | **KE+CHO** | **CHO** | **KE+CHO** | **CHO** | **KE+CHO** | **CHO** | **KE+CHO** | **CHO** | **KE+CHO** | **CHO** | **KE+CHO** |
| **Glucose** *(mmol/L)* | 5.2  [4.6-6.1] | 6.1  [5.0-7.1] | 9.2  [8.5-11.3] | 7.3  [4.9-8.9] | 8.3  [7.5-11.3] | 6.3  [4.4-7.6] | 7.9  [3.7-8.9] | 4.8  [4.5-5.9] | 6.8  [4.0-8.5] | 4.7  [4.3-5.6] | 6.4  [4.8-7.4] | 6.4  [5.8-7.4] |
| **βHB**  *(mmol/L)* | 0.03  [0.02-0.41] | 0.05  [0.02-0.74] | 0.02  [0.01-0.08] | 2.56  [1.57-2.88] | 0.03  [0.01-0.08] | 2.29  [1.31-2.72] | 0.02  [0.01-0.04] | 2.36  [1.35-2.94] | 0.04  [0.02-0.08] | 1.99  [1.01-3.13] | 0.03  [0.03-0.26] | 0.11  [0.06-0.82] |
| **AcAc**  *(mmol/L)* | 0.04  [0.02-0.18] | 0.04  [0.03-0.31] | 0.02  [0.01-0.04] | 0.79  [0.67-1.24] | 0.02  [0.01-0.04] | 0.88  [0.61-1.09] | 0.02  [0.01-0.02] | 1.00  [0.52-1.12] | 0.02  [0.02-0.09] | 0.92  [0.05-1.24] | 0.05  [0.24-0.03] | 0.05  [0.05-0.39] |
| **FFA**  *(μmol/L)* | 62  [25-636] | 84  [53-310] | 68  [31-300] | 90  [72-154] | 79  [29-223] | 74  [64-134] | 55  [19-319] | 51  [35-86] | 54  [12-551] | 35  [31-107] | 531  [71-1018] | 100  [58-836] |
| **Lactate**  *(mmol/L)* | 1.8  [1.0-2.9] | 2.0  [0.8-3.1] | 2.3  [2.0-2.8] | 2.5  [1.8-3.1] | 2.7  [1.9-3.5] | 2.8  [2.0-3.1] | 2.8  [1.2-4.1] | 2.8  [1.4-3.5] | 2.7  [1.1-4.4] | 2.5  [1.6-3.3] | 1.4  [0.8-1.9] | 1.9  [1.0-2.4] |
| **Insulin**  *(mU/L)* | 19  [7-47] | 19  [3-45] | 114  [38-224] | 74  [28-119] | 68  [24-139] | 39  [16-85] | 47  [6-95] | 19  [8-39] | 36  [1.5-76] | 14  [7-22] | 20  [6-41] | 26  [9.5-53] |

**Table S1.** Changes in blood metabolites after ingestion of CHO (arm A) and KE+CHO (arm B) study drink from all subjects (n=6).

Legend: Values expressed as median [range].

|  | **CHO** | | | **KE+CHO** | | |
| --- | --- | --- | --- | --- | --- | --- |
|  | *Rest* | *30%* | *60%* | *Rest* | *30%* | *60%* |
| **#1** | 0.97 | 0.82 | 0.94 | 1.00 | 0.93 | 0.94 |
| **#2** | 1.20 | 1.14 | 0.99 | 1.05 | 1.00 | 0.91 |
| **#3** | 0.91 | 0.90 | 1.16 | 1.04 | 0.95 | 1.00 |
| **#4** | 0.94 | 0.81 | 0.96 | 1.02 | 0.90 | 0.93 |
| **#5** | 0.95 | 0.97 | 1.13 | 0.96 | 0.85 | 1.04 |
| **#6** | 1.05 | 0.92 | 1.02 | 1.00 | 0.82 | 0.94 |

**Table S2.** Individual data on RER during the upright bicycle protocol in both study arms.

Legend: Values expressed as mean±SD

|  | **CHO** | | **KE+CHO** | |
| --- | --- | --- | --- | --- |
|  | *30%* | *60%* | *30%* | *60%* |
| **#1** | 7.2 | 7.2 | 7.1 | 7.1 |
| **#4** | 7.2 | 7.1 | 7.0 | 7.1 |
| **#5** | - | - | 7.0 | 7.0 |
| **#6** | 7.0 | 7.0 | 7.1 | 7.1 |

**Table S3.** Individual data on muscle pH at 30% and 60% Wmax equivalents in both study arms.

|  | **CHO** | | **KE+CHO** | |
| --- | --- | --- | --- | --- |
|  | *Rest* | *End exercise^1^*  *(162 sec)* | *162 sec* | *End exercise^1^*  *(229 sec)* |
| **#3** | 7.1 | 6.9 | 7.0 | 7.1 |

**Table S4.** Muscle pH in both study arms during exercise in subject #3.

Legend: ^1^The in vivo 31P MR spectra recorded from the quadriceps muscle at exhaustion in each study arm were best explained by two distinct populations of contracting fibers operating at cytosolic pH values of 6.9 and 7.6, respectively (CHO arm) versus 7.1 and 8.1, respectively (KE+CHO arm).
